# Patient-Reported Outcomes and Preferences for Total Hip Arthroplasty Approach—Crossover Cohort Study

**DOI:** 10.3390/jcm15031127

**Published:** 2026-01-31

**Authors:** Paweł Skowronek, Paweł Jankowski, Katarzyna Czarzasta, Mateusz Kawka

**Affiliations:** 1 Department of Mini-Invasive Orthopedics and Rehabilitation, Medical University of Warsaw, Żwirki i Wigury 61 Str., 02-091 Warsaw, Poland; 2 Department of Orthopedic Surgery and Rehabilitation, Bródnowski Hospital of Mazovian Region, Kondratowicza 8 Str., 03-242 Warsaw, Poland; 3 Department of Experimental and Clinical Physiology, Laboratory of Center for Preclinical Research, Medical University of Warsaw, Banacha 1B Str., 02-097 Warsaw, Poland

**Keywords:** total hip arthroplasty, direct anterior approach, posterolateral approach, lateral approach

## Abstract

**Background:** Evidence comparing the Direct Anterior Approach (DAA) for total hip arthroplasty (THA) to other approaches is conflicting, particularly regarding patient-reported outcomes. This study aimed to compare the outcomes and preferences of the DAA and Posterolateral (PLA) and Lateral (LA) Approaches within a crossover cohort. **Methods:** This retrospective crossover study included 69 patients who underwent staged bilateral THA with a DAA on one hip and either a PLA (*n* = 29) or LA (*n* = 40) on the contralateral hip. At a minimum 12-month follow-up, patient-reported outcomes, including length of stay, mobilization, crutch use, functional scores (mHHS, HOOS-PS, and NRPS), and preferences, were collected via telephone survey and analyzed using a paired *t*-test. **Results:** Compared to other approaches, the DAA resulted in a significantly shorter length of stay (*p* < 0.001), earlier mobilization (*p* < 0.001), and shorter duration of crutch use (*p* < 0.001). At 12 months, the DAA group also reported higher modified Harris Hip Scores (*p* < 0.05) and lower pain scores (*p* < 0.05). The majority of patients preferred DAA to both PLA (60.7%) and LA (72.5%). **Conclusions:** In this within-patient comparison, DAA provided a significantly faster early recovery and was strongly preferred by patients. These early postoperative advantages are critical for patients, and should be prominent in the shared decision-making process for THA.

## 1. Introduction

Total hip arthroplasty (THA), also known as “the operation of the century,” is one of the most successfully implemented operative procedures, enabling patients with advanced hip osteoarthritis to recover quickly with a high rate of satisfaction [1]. The global burden of osteoarthritis is significant and continues to increase, driven by an aging population and rising obesity levels. In 2020, an estimated 595 million people were living with osteoarthritis, a figure projected to approach one billion by 2050 [2,3]. Specifically, the prevalence of hip osteoarthritis is a major contributor to this burden, creating an unprecedented demand for effective treatment.

This escalating epidemiological trend is placing immense pressure on the healthcare systems worldwide. In the United States alone, the annual number of primary THA procedures is projected to increase by 284%, reaching over 1.4 million operations by 2040 [4]. This dramatic increase in volume generates a significant economic challenge, compelling a focus on value-based care and cost optimization throughout the entire episode of care. Moreover, as revision surgeries are substantially costlier than primary procedures, selecting the optimal approach for an implant combination from the outset is essential for both patient outcomes and healthcare sustainability [5,6,7,8].

The surgical approach to the hip is a key variable that affects the recovery, function, and complication rates. The three most common approaches are the Posterolateral, Lateral, and Direct Anterior Approach (DAA). The Posterolateral Approach, the most widely used globally, offers excellent exposure but involves division of the short external rotator muscles and capsule, which has historically been associated with a higher risk of postoperative dislocation [9]. The Lateral Approach provides robust stability but can involve dissection of the abductor muscles (gluteus medius and minimus), potentially leading to a temporary or, in rare cases, persistent limp [9].

In contrast, DAA has gained significant popularity over the past two decades. First described by the German surgeon Carl Hueter and later popularized in North America by Smith-Petersen for other hip procedures, this technique accessing the hip joint between the tensor fasciae latae and sartorius muscles superficially, and the gluteus medius and rectus femoris deeply. Unlike PLA or LA, this truly intermuscular and internervous plane does not require the detachment or splitting of muscles, which theoretically preserves dynamic hip stability and reduces postoperative pain, facilitating earlier mobilization [10]. The reported advantages of this tissue-sparing technique include the potential for less postoperative pain, accelerated early functional recovery, shorter hospital stay, and a lower risk of dislocation [5,11,12].

Despite the theoretical benefits of the DAA, the existing body of literature presents conflicting results, with many systematic reviews and meta-analyses failing to show a significant long-term advantage over other approaches [13,14,15]. Furthermore, a critical gap exists in the current evidence: there is a lack of high-quality studies that rigorously control for inter-patient variability, taking into account the personal experiences and preferences of patients. Most comparative studies rely on clinician-reported data or patient-reported outcome measures (PROMs) from separate patient groups, which can be confounded by patient-specific factors such as pain tolerance, motivation, and baseline function. A crossover study design, in which the same patient underwent THA using a different approach on each hip, offers a unique and powerful methodology to eliminate these confounding variables and provide an accurate within-patient comparison [12]. Such a design may be crucial for discerning essential differences in outcomes and for capturing patient preferences.

Therefore, this study aimed to retrospectively compare patient-reported outcomes and preferences for total hip arthroplasty performed using different surgical approaches in a crossover patient cohort.

## 2. Materials and Methods

This was a retrospective, single-center crossover cohort study. This study analyzed a cohort of 69 patients who underwent staged bilateral total hip arthroplasty (THA), with each procedure performed through a different surgical approach: Direct Anterior (DAA), Posterolateral (PLA), and Lateral (LA). Two cohorts of patients were formed: (1) patients who underwent bilateral staged THA operated first through PLA and DAA, and (2) patients who underwent bilateral staged THA operated first through the LA and DAA.

The decision to utilize a different approach for the second hip was driven primarily by the evolution of surgical expertise and the shift in the preferred operative technique within our department. Over the study period (2002–2024), as specialized proficiency in the Direct Anterior Approach was established, it replaced PLA/LA as the standard of care for primary THA. Consequently, patients who had their first hip operated on in the earlier years via PLA or LA underwent their contralateral procedure via DAA in later years, reflecting the progression of surgical specialization.

All THA procedures were performed by an experienced surgeon highly specialized in the respective surgical approach. Informed consent was obtained from all the participants included in this study.

The inclusion criteria were as follows: (i) patients who underwent staged, bilateral primary THA, (ii) utilization of two different surgical approaches with PLA or LA as the prior and DAA as the subsequent, (iii) a minimal time interval between surgeries of 6 months, and (iv) a minimum follow-up period of 12 months after the second procedure. The exclusion criteria included incomplete follow-up data or loss of contact with the patient.

All patients were managed using a standardized perioperative protocol. The protocol included adequate preoperative patient education. Admission to the hospital occurred 24 h before the planned procedure. All patients were treated with spinal anesthesia and multimodal postoperative analgesia. Since the day of the operation, patients were administered low-molecular-weight heparin as prophylaxis for venous thromboembolism, with early mobilization by a physical therapist on the first postoperative day.

Standardized protocols were used for all surgical approaches without intraoperative fluoroscopy. Patients who underwent the Direct Anterior Approach (DAA) were positioned supine on a standard surgical table. The hip joint was accessed through the intermuscular and internervous planes as described previously. Patients were placed in the lateral decubitus position using the Posterolateral Approach (PLA). The hip joint was accessed by splitting the gluteus maximus muscle and detaching the short external rotator muscles from the femoral insertions. For the Lateral Approach (LA), patients were also positioned in the lateral decubitus position. Access to the hip was gained through an interval involving the gluteus medius and the minimus muscles. Following the procedure, the patients were instructed to avoid hip flexion beyond 90°, adduction, and internal rotation for the first six weeks.

Demographic data and subjective functional outcomes (mHHS, HOOS-PS, NRPS, preferences) were collected via telephone survey. However, objective clinical metrics, including the exact Length of Stay (LOS), complications, reoperation rates, and the specific dates of surgeries, were verified against the hospital’s electronic medical records to ensure accuracy and eliminate recall bias for these parameters.

Patient-Reported Outcome Measures (PROMs) using validated questionnaires were used to evaluate functional outcomes and quality of life. The patients completed the surveys postoperatively after a 12-month follow-up period. The scales included the modified Harris Hip Score (mHHS), hip disability and osteoarthritis outcome score (HOOS-PS), and Numeric Pain Rating Scale (NRPS). Patient preferences regarding the surgical approach were gathered through direct questions during the telephone survey.

Statistical analysis was performed using Statistica 14™ software (Tibco^®^, Palo Alto, CA, USA). Continuous variables are presented as means and standard deviations (SD), while categorical variables are presented as counts and percentages. A paired *t*-test was used to compare continuous variables between specific pairs of approaches in the same patient. Categorical variables (e.g., patient preference and complication rates) were examined using the chi-square test. The threshold for statistical significance was set at *p* < 0.05.

## 3. Results

A total of 69 patients were included in the final analysis, comprising 40 women (58%) and 29 men (42%). Patients underwent staged bilateral total hip arthroplasty, with the second procedure performed via the Direct Anterior Approach (DAA) and the initial contralateral hip via PLA (29 patients) or LA (40 patients). The demographic and baseline clinical characteristics of each surgical group are shown in Table 1. All procedures were conducted between 2002 and 2024.

The mean age of the patients at the time of the DAA procedure was 68.3 years (range: 30–89), which was higher than the mean age of the PLA at 61.6 years (range: 36–80) and LA at 62.5 years (range: 29–84). The mean Body Mass Index (BMI) was similar across the groups, with 30.1 kg/m^2^ for the DAA group, 29.8 kg/m^2^ for the PLA group, and 28.7 kg/m^2^ for the LA group.

A detailed comparison of the clinical outcomes between the groups is shown in Table 2.

**Figure 1:**
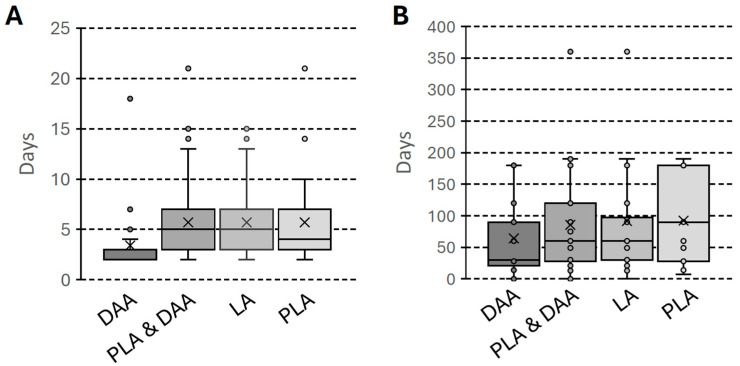
Box-and-whisker plot comparing the Length of Stay (LOS) (**A**) and number of days of crutch walking (**B**) following total hip arthroplasty in days via Direct Anterior Approach (DAA), Posterolateral Approach (PLA), and Lateral Approach (LA). The horizontal line within each box represents the median, while the top and bottom of the box indicate the interquartile range (25th to 75th percentiles). The whiskers extend to the minimum and maximum values excluding outliers, which are plotted as individual points.

Statistically significant differences were observed in the key early postoperative indicators, favoring the DAA. The length of stay was notably shorter in the DAA group, with an average of 3.39 ± 2.27 days compared to 5.70 ± 3.66 days in the PLA/LA group (*p* < 0.001), as illustrated in Figure 1A. Patients in the DAA group also mobilized significantly earlier, at an average of 1.43 ± 0.50 days post-operation, whereas this period was 2.10 ± 1.32 days for the PLA/LA group (*p* < 0.001).

Statistically significant differences were also observed when assessing functional outcomes and pain. The mean modified Harris Hip Score (mHHS) at 12 months was notably higher in the DAA group, reaching 73.68 ± 13.49 points compared to 69.43 ± 18.80 points in the PLA/LA group (*p* < 0.05). This was accompanied by significantly lower pain in the DAA group, with a mean NRPS score of 1.59 ± 1.95 versus 2.26 ± 2.62 in the PLA/LA group (*p* < 0.05). The only parameter that did not show a significant difference was the HOOS-PS questionnaire score, which measures physical function (DAA: 14.99 ± 3.78 vs. PLA/LA: 14.80 ± 4.10; *p* > 0.05).

The analysis also showed that patients who underwent the DAA procedure stopped using crutches more quickly. The average time of walking with crutches in this group was 68.32 ± 56.62 days, significantly less than the PLA/LA group, where patients needed an average of 101.75 ± 81.99 days (*p* < 0.001). The distribution of time required for walking with crutches across the groups is presented in Figure 1B.

In a more precise comparison between the Direct Anterior Approach (DAA) and the Posterolateral Approach (PLA), statistically significant differences favoring DAA were found regarding recovery metrics (LOS, mobilization, crutch use). Detailed comparisons between the DAA and PLA groups are shown in Table 3.

The average length of stay was notably shorter for the DAA group at 3.07 ± 1.10 days, compared to 5.69 ± 4.30 days for the PLA group (*p* < 0.05), as illustrated in Figure 1A. Likewise, the day of mobilization was significantly earlier for DAA patients, happening at an average of 1.55 ± 0.51 days after surgery compared to 2.34 ± 1.76 days for PLA patients (*p* < 0.05).

The functional and pain assessments also showed significant differences. The modified Harris Hip Score (mHHS) was higher in the DAA group (73.07 ± 15.13) compared to the PLA group (70.00 ± 18.39), but this difference did not reach statistical significance (*p* > 0.05). Pain scores on the NRPS were comparable between the groups (DAA: 2.28 ± 2.16 vs. PLA: 2.17 ± 2.98; *p* > 0.05). Another outcome without a statistically significant difference was the HOOS-PS score (DAA: 14.69 ± 4.64 vs. PLA: 14.79 ± 4.61; *p* > 0.05).

Finally, the recovery milestone for discontinuing assistive devices was much faster in the DAA group. The mean time of walking with crutches was 72.96 ± 58.16 days for DAA patients, which was significantly shorter than the 100.48 ± 62.79 days required for PLA patients (*p* < 0.05), as illustrated in Figure 1B.

When comparing the Direct Anterior Approach (DAA) to the Lateral Approach (LA), the DAA group demonstrated significantly shorter recovery times, although the differences in functional scores were less pronounced. A detailed summary is presented in Table 4.

The mean length of stay was significantly shorter for DAA patients at 3.63 ± 2.83 days, compared to 5.70 ± 3.16 days for the LA group (*p* < 0.001), as illustrated in Figure 1A. Similarly, the day of mobilization was significantly earlier in the DAA group, occurring at a mean of 1.34 ± 0.48 days versus 1.92 ± 0.82 days for the LA group (*p* < 0.001). The most notable difference was in the time of walking with crutches, which was significantly shorter for the DAA group at 64.91 ± 56.08 days, compared to 102.68 ± 94.59 days in the LA group (*p* < 0.001).

In the assessment of functional outcomes and pain, no statistically significant differences were found between the two approaches. The mean modified Harris Hip Score (mHHS) was 74.13 ± 12.35 for the DAA group and 69.03 ± 19.32 for the LA group (*p* < 0.05). Pain levels on the NRPS were also comparable (DAA: 1.09 ± 1.64 vs. LA: 2.33 ± 2.36; *p* < 0.05), as were the HOOS-PS scores (DAA: 15.20 ± 3.06 vs. LA: 14.80 ± 3.76; *p* > 0.05).

A telephone survey was conducted to assess the patients’ subjective preferences regarding the surgical approach they received. The results were analyzed separately for each group. In a cohort of patients comparing the Direct Anterior Approach (DAA) with the Posterolateral Approach (PLA), the majority of patients indicated a preference for DAA. Of the 27 patients in this group, 17 (60.7%) preferred DAA, while 8 (28.6%) chose PLA. Three patients (10.7%) reported no preference, considering both approaches comparable.

**Figure 2:**
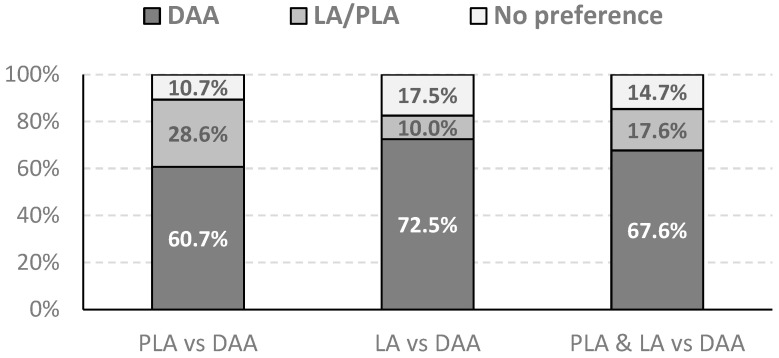
Patient preferences regarding the surgical approach in the crossover cohorts. The charts depict the percentage of patients preferring the Direct Anterior Approach (DAA) versus the Posterolateral Approach (PLA) and the DAA versus the Lateral Approach (LA). A clear majority of patients favored the DAA in both comparison groups (60.7% vs. PLA and 72.5% vs. LA), indicating a strong subjective preference for the anterior technique.

A similar, even more pronounced trend was observed in the group comparing the DAA with the Lateral Approach (LA). In this cohort of 40 patients, 29 (72.5%) reported a preference for DAA. LA was preferred by four patients (10%), and seven patients (17.5%) had no specific preference. The comparison of patient preferences in both cohorts is depicted in Figure 2.

## 4. Discussion

The primary finding of this study is that the Direct Anterior Approach (DAA) is associated with a significantly faster early recovery and is overwhelmingly preferred by patients in a crossover cohort compared to both the Posterolateral (PLA) and Lateral (LA) Approaches for total hip arthroplasty (THA). Our results provide strong, patient-centered evidence that contributes to the ongoing debate regarding the optimal surgical approach for THA. By using a crossover design, where each patient served as their own control, we were able to minimize the confounding effects of inter-patient variability, which is a significant limitation in many comparative orthopedic studies.

Our observation of a shorter length of stay, earlier mobilization, and more rapid discontinuation of walking aids in the DAA group aligns with a substantial body of the literature. Several studies have reported similar accelerated recovery metrics for DAA, which is attributed to its muscle-sparing nature [5,12]. In our direct comparison with the PLA group, the DAA group also demonstrated superior early functional scores (mHHS) and lower pain levels (NRPS). However, it is noteworthy that these functional and pain score advantages were not statistically significant compared with LA. This nuanced finding suggests that, while the recovery advantages of DAA are robust, the differences in patient-reported function may vary depending on the specific alternative approach. Furthermore, as Malek et al. demonstrated, implementing a comprehensive Enhanced Recovery After Surgery (ERAS) pathway can significantly mitigate differences in early outcomes between approaches, suggesting that the surgical technique is just one component of successful recovery [16].

The most compelling finding of our study was the strong patient preference for DAA. In both comparison cohorts, a clear majority of patients favored DAA and reported that their recovery was easier. This aligns perfectly with the findings of Rhea et al., who also utilized a crossover design and found that 70% of patients preferred DAA over PLA [12]. However, our results stand in contrast to the study by Radoicic et al., which, despite a similar methodology, found that 76% of their patients preferred the posterior approach [17]. This discrepancy in patient preference across studies suggests that patient experiences are multifactorial. It may be influenced by factors beyond objective clinical outcomes, including preoperative expectations, information from friends or media, and the perceived “modernity” of a technique, as highlighted by Trousdale et al. [18].

The primary strength of our study was its crossover design, which eliminated inter-patient variability by having each patient serve as their own control. This is a robust methodology for assessing subjective outcomes and preferences [12,19]. An additional advantage is the inclusion of two distinct comparison groups, allowing the evaluation of the DAA against both the Posterolateral and Lateral Approaches. This structure provides a more comprehensive and nuanced picture, demonstrating that the early postoperative advantages of DAA are consistent when compared to both of the most common alternatives. However, our study had several limitations. Its retrospective nature introduces a risk of recall bias [20]. We acknowledge that the extended timeframe of the study (spanning over 20 years) introduces a potential confounding variable related to perioperative care improvements. The Direct Anterior Approach (DAA) procedures were performed more recently and likely benefited from modern Enhanced Recovery After Surgery (ERAS) protocols, which were not fully established during the era of the earlier PLA/LA surgeries. Therefore, the superior early recovery metrics observed in the DAA group may reflect the synergistic effect of the tissue-sparing approach and modern perioperative management.

Furthermore, the crossover design inherently places the DAA as the second procedure. Some authors suggest that recovery after second hip surgery might be perceived as easier due to the elimination of contralateral hip pain or patient familiarity with the rehabilitation process. However, other studies indicate that patients often judge the second surgery more critically or suffer from ‘recency bias,’ forgetting the pain severity of the first remote surgery [21]. By focusing on comparative preference, we aimed to capture the patient’s holistic evaluation of the experience, regardless of these temporal factors. Finally, this was a single-center study, which may have limited the generalizability of our findings.

Future research should build upon these findings with prospective, randomized crossover trials, in which the order of the surgical approaches is randomized to minimize any carryover effect. Incorporating objective activity measures, such as data from accelerometers, would also be valuable in verifying whether patients’ subjective feelings of faster recovery are supported by their actual daily physical activity. Finally, long-term follow-up is necessary to determine whether the early benefits and patient preferences observed in our study are sustained over time.

## 5. Conclusions

In conclusion, this study provides additional within-patient evidence that the DAA offers a faster early recovery and is highly preferred by patients compared to the Posterolateral and Lateral Approaches. Although long-term functional outcomes may converge, the significant advantages of early postoperative experience are a critical factor for patients. This should be a key consideration in the shared decision-making process for total hip arthroplasty.

## Figures and Tables

**Table 1:** Characteristics of patient groups.

	DAA	PLA/LA	PLA	LA
Year of operation (range)	2013–2024	2002–2023	2002–2022	2004–2023
Age (range)	30–89	29–84	36–80	29–84
Mean age	68.3	62.1	61.6	62.5
Median age	70	63	63	63.5
Number of patients	69	69	29	40
Mean length of stay	3.4	5.7	5.7	5.7
Median length of stay	3	5	4	5
Number of revisions	3	7	4	3
Revisions rate	4%	10%	14%	8%
Number of preferences	46	13	8	5
Preference rate	67%	19%	28%	13%

**Table 2:** Comparison between the DAA and PLA/LA groups.

	DAA	PLA/LA	*p*
	Mean (±SD)	Mean (±SD)	
Length of stay	3.39 ± 2.27	5.70 ± 3.66	*p* < 0.001
Day of pionization	1.43 ± 0.50	2.10 ± 1.32	*p* < 0.001
mHHS	73.68 ± 13.49	69.43 ± 18.80	*p* < 0.05
NRPS	1.59 ± 1.95	2.26 ± 2.62	*p* < 0.05
HOOS-PS	14.99 ± 3.78	14.80 ± 4.10	*p* > 0.05
Days of crutch walking	68.32 ± 56.62	101.75 ± 81.99	*p* < 0.001

**Table 3:** Comparison between DAA and PLA groups.

	DAA	PLA	*p*
	Mean (±SD)	Mean (±SD)	
Length of stay	3.07 ± 1.10	5.69 ± 4.30	*p* < 0.05
Day of pionization	1.55 ± 0.51	2.34 ± 1.76	*p* < 0.05
mHHS	73.07 ± 15.13	70.00 ± 18.39	*p* > 0.05
NRPS	2.28 ± 2.16	2.17 ± 2.98	*p* > 0.05
HOOS-PS	14.69 ± 4.64	14.79 ± 4.61	*p* > 0.05
Days of crutch walking	72.96 ± 58.16	100.48 ± 62.79	*p* < 0.05

**Table 4:** Comparison between DAA and LA groups.

	DAA	LA	*p*
	Mean (±SD)	Mean (±SD)	
Length of stay	3.63 ± 2.83	5.70 ± 3.16	*p* < 0.001
Day of pionization	1.34 ± 0.48	1.92 ± 0.82	*p* < 0.001
mHHS	74.13 ± 12.35	69.03 ± 19.32	*p* < 0.05
NRPS	1.09 ± 1.64	2.33 ± 2.36	*p* < 0.05
HOOS-PS	15.20 ± 3.06	14.80 ± 3.76	*p* > 0.05
Days of crutch walking	64.91 ± 56.08	102.68 ± 94.59	*p* < 0.001

## Data Availability

The data presented in this study are available on request from the corresponding author. The data are not publicly available due to privacy and ethical restrictions regarding patient confidentiality.

## Abbreviations

The following abbreviations are used in this manuscript:

DAA	Direct Anterior Approach
PLA	Posterolateral Approach
LA	Lateral Approach
THA	Total Hip Arthroplasty
mHHS	modified Harris Hip Score
NRPS	Numeric Pain Rating Score
HOOS-PS	Hip disability and Osteoarthritis Outcome Score—Physical Function Shortform
